# The role of connected diagnostics in strengthening regional, national and continental African disease surveillance

**DOI:** 10.4102/ajlm.v7i2.775

**Published:** 2018-12-06

**Authors:** Natasha M. Gous, Philip C. Onyebujoh, Alash’le Abimiku, Chris Macek, Jeff Takle

**Affiliations:** 1 SystemOne, LLC, Johannesburg, South Africa; 2 SystemOne, LLC, Springfield, Massachusetts, United States; 3 Africa Centres for Disease Control and Prevention, African Union, Addis Ababa, Ethiopia; 4 International Research Center of Excellence, Institute of Human Virology, Abuja, Nigeria

## Summary

The Africa Centres for Disease Control and Prevention (Africa CDC) and World Health Organization (WHO) Regional Office for Africa (AFRO) are building a global health security programme that aims to strengthen both regional and national health through networked, collaborative efforts to improve infectious disease and antimicrobial resistance surveillance. To achieve this, the Africa CDC is calling for a data-sharing platform that can be leveraged across member states and disease areas, strengthening the ability to collate, analyse and interpret data, and to respond with the appropriate action.

Although numerous disease intelligence and surveillance systems exist, they are plagued with inaccurate or untimely data. We contend, furthermore, that it was this lack of data *quality* – and not the lack of surveillance systems or networks – that prevented the global community from acting earlier in response to the Ebola outbreak in 2014–2016. The new field of ‘connected diagnostics’ is one solution to this concern, as it automates data collection directly from the diagnostic instruments to multiple levels of stakeholders for real-time decision-making and policy response.

This article details how the intervention of ‘connected diagnostics’ could solve the primary underlying failure in existing surveillance systems – *the lack of accurate and timely data* – to enable difficult political decisions earlier. The use of connectivity solutions can enable critical health and operational data to empower the Africa CDC, regional hubs, and each country with a consistent and automated data feed while still maintaining country privacy and controls.

## Background

In an interconnected and interdependent world, the threat of any infectious disease is no longer simply a national health concern. Globalisation, despite all of its advantages, has resulted in a world where infectious pathogens can effortlessly traverse borders and threaten global public health security. Outbreaks of infectious diseases are increasing globally with a resurgence of epidemics such as cholera, Ebola and dengue, particularly in Africa. So too, the rise of antimicrobial resistance (AMR) partly due to the increasing use of antibiotics, particularly in low-income countries, poses a worldwide biosecurity and economic threat. The number of deaths globally due to AMR is projected to reach a staggering 10 million per year by 2050.^1^

The requirement for timely reporting of infectious diseases has been highlighted on several occasions. A poignant reminder of the impact of poor data quality and timeliness in the management of disease outbreaks is the Ebola outbreak in 2014–2016 – an outbreak that resulted in the loss of $2.2 billion US dollars gross domestic product in Guinea, Sierra Leone and Liberia^2^ as well as 11 325 lives.^3^ In 2015, an outbreak of a ‘strange disease’ occurred in Kampala, Uganda. The outbreak was thought to have begun in January 2015 or earlier, but was not recognised and reported as a typhoid outbreak until February 2015 due to the lack of surveillance systems; this delay led to thousands being affected and several deaths.^4^ Some countries are reporting an average delay of 22 days from collection of infectious disease specimens to return of results to facilities.^5^ Similarly, in the context of HIV, the return of laboratory result to referring facilities can take up to 90 days^6^ with up to 50% loss of results,^7^ thus, severely delaying initiation of treatment, adherence counselling and switch to second-line treatment.

Monitoring of infectious diseases and AMR through surveillance systems is the cornerstone of any outbreak and response network and the availability of a robust and timely system will ensure a country’s propensity to manage and promptly contain an outbreak.^8^ Time, more than anything, determines the success of a response. However, surveillance systems are largely fragmented and lack input of timely, accurate data.

### Disease surveillance systems

Disease surveillance systems serve two key functions: first, to provide an early warning system for potential threats and, second, to provide a system to monitor diseases, trends, and progress towards control and elimination strategies.^9^ Worldwide, different disease surveillance strategies and systems have been adopted. Traditional systems typically rely on a notification-based approach, whereby health care providers regularly submit data to relevant health authorities on a specified infectious disease within a specified territory.^8^ This requires passive reporting on disease information and, although inexpensive, can result in sequential errors, reflecting under- and over-reporting by health care providers of diseases and cases. Active surveillance systems, on the other hand, rely on proactive searches for cases with established criteria, risk factors or events, but still require manual data collection. By design, both of these strategies, although employing structured reporting mechanisms, suffer numerous challenges, including the lack of or inaccessibility of real-time, quality data, under-reporting or delayed reporting of cases and deaths,^8^ and complex management.^10^

Advancements such as the Internet have revolutionised how disease data is collected and now, Web-based surveillance systems such as Healthmap, allow automated and rapid collection of large amounts of unstructured data from electronic sources.^10,11,12^ However, even with advancements, Web-based surveillance is still challenging, as the information collected from diverse sources is not structured,^12^ has uncertain and varying data quality, and may lead to inaccurate interpretation and predictions.^10^

## Disease surveillance in Africa

Africa has been plagued by numerous and recurring epidemics over the past several decades. Not only do these diseases share the ability to decimate entire towns and villages, they also place enormous economic strain on countries.^13^ Global estimates by the World Bank place the annual global cost of a moderately severe to severe pandemic at roughly $570 billion.^14^ Responses to many of these diseases have been hampered by weak health care systems, lack of policies that encourage integration and coordination within countries and across borders, and the absence of accurate and timely diagnostic data for decision-making.

When the WHO AFRO attempted to provide an inventory of all epidemics reported in Africa between 1970 and 2016, the effort was impeded by limited data, inconsistencies in reporting of occurrences and magnitudes of outbreaks, and variability in description of outbreak locations.^15^

The increasing burden of AMR threatens the effectiveness and success of infectious disease treatment programmes.^16^ Africa is thought to contribute a large proportion of the global AMR burden due to limited control and monitoring of use of antibiotics and the high rates of communicable diseases such as HIV and tuberculosis.^17^ However, the scarcity of data coming from the region on AMR surveillance reflects the absence of tools to collect valuable information, reliance on passive reporting from centres and lack of training and expertise needed for continuous monitoring and reporting.^16^ One review on the status of AMR in Africa found that recent data on AMR was not available for 42.6% of African countries and what was available were lacking in quality, even though resistance to commonly prescribed antibiotics was very high in the African continent.^18^ A review of 135 current antibiotic prescribing guidelines also found that, in general, most do not consider resistance patterns for highly prevalent infectious syndromes such as community-acquired pneumonia and urinary tract infections.^19^ This largely reflects the lack of collection of accurate resistance data and limits our true understanding of AMR burden.

### The role of the Africa Centres for Disease Control and Prevention in antimicrobial drug resistance surveillance

To strengthen both regional and national health, the Africa CDC and WHO AFRO are building a global health security programme, focusing on rapid response surge teams, starting at the Africa CDC and stemming out to the national public health institutes (NPHIs). Five regional collaborating centres (RCCs) hosted by Zambia, Kenya, Gabon, Nigeria and Senegal, in addition to key NPHIs in those sub-regions, make up the Africa CDC Regional Integrated Surveillance Laboratory Network or RISLNET,^20^ with headquarters in Ethiopia. The goal of this integrated system is to drive networked, collaborative efforts to strengthen and improve disease surveillance linked to laboratory confirmation, disease preparedness and response. A further expected outcome of the Africa CDC is the establishment of the Antimicrobial Resistance and Surveillance Network or AMRSNET that will standardise the approach to AMR surveillance and strive to achieve quality data.^20^ In order for both of these efforts to be fruitful, a data-sharing platform is needed which can be leveraged across member states and disease areas to strengthen the ability to collate, analyse and interpret the generated data, and to respond appropriately.

## Connected diagnostics in disease surveillance

To ensure an effective response to a potential outbreak event, timeliness is of paramount importance. This requires surveillance, assessment and communication mechanisms to be in place to increase awareness of management strategies, and to facilitate their initiation in the early phases.^21,22^ When a passive, paper-based surveillance system was implemented in the public health sector in South Africa for health care-associated infections, the main challenge was incomplete data collection.^23^ Nurses felt that manual collection and recording of data added to their workload, thus highlighting the need for systems that are able to automatically collect data and communicate it to various levels of the health care system. Interconnected diagnostic networks can address these needs by facilitating the interactions between laboratory confirmation, automated data collection, interpretation, delivery of results and real-time monitoring of disease as well as analysis. Also, by providing accurate and reliable diagnostic information directly to the point of patient care, more timely patient management and appropriate use of antimicrobials can be facilitated.

### Real-time reporting of laboratory-confirmed cases

A critical component of disease-specific surveillance and early warning systems is their reliance on laboratory confirmation of the disease. High-quality, reliable laboratory detection has been emphasised by the Africa CDC as the central component to rapid response.^20^

With the evolution of medical diagnostic instruments and availability of digital health platforms in many African countries, the tools for detection and response are already in place and ready to be used and connected for surveillance. As an example, the GeneXpert platform (Cepheid, Sunnyvale, California, United States) is a molecular diagnostic tool commonly used for tuberculosis and first-line drug resistance detection, but also allows detection of a range of other diseases including HIV, Hepatitis C virus, Ebola, Methicillin-resistant *Staphylococcus aureus* and flu through the use of pathogen-specific cartridges. The platform produces a wealth of electronic diagnostic and operational data that can be collected and transmitted in real-time, directly from the instrument, utilising connectivity solutions such as the GxAlert/Aspect platform (SystemOne, LLC, Springfield, Massachusetts, United States) or C360 (Cepheid, Sunnyvale, California, United States).^24^ In the context of tuberculosis, the move towards connected diagnostic platforms has been strongly recommended by the WHO in the latest Global Laboratory Initiative guideline on connectivity and as part of the End TB Strategy. The guideline indicates that all sites that use WHO-recommended rapid tuberculosis diagnostics should be transmitting results electronically to clinicians and to information management systems, using data connectivity solutions by no later than 2020.^24^

Currently, the existing GeneXpert footprint in Africa is extensive; the WHO AFRO accounted for 42% of the global GeneXpert module procurement in 2016 and almost 65% of total cartridge procurement.^25^ By leveraging this existing footprint, connected GeneXpert systems could shorten the detection-response gap, reduce patient loss to follow-up and facilitate early antimicrobial therapy and thus reducing morbidity and mortality.^26^

### Automated alerts and triggers

The paper-based systems still found in many nations today have notoriously slow reporting cycles. Disease surveillance in a NPHI may consist of manual tabulation of vital registries and death certificates. These sources are of questionable accuracy (e.g. cause of death listed as ‘fever’ instead of ‘Ebola infection’) and typically tabulated on an annual or quarterly basis, which is far too slow to identify an outbreak in progress and prevent it from reaching critical mass.

Lack of knowledge regarding disease thresholds has also been found to greatly restrict early identification of disease outbreaks.^27^ Connectivity solutions can assist by providing an in-built system to trigger automated electronic alerts when reported cases of a specific disease exceed a predefined threshold. This trigger could be sent to an epidemiologist for specialist analysis or to field staff to verify the occurrence of outbreaks and ensure that prompt control measures and case findings are instituted. Setting these thresholds is a crucial component to early warning and outbreak systems and needs to be based on characteristics of the local disease.^28^ During the 2014–2016 Ebola outbreak in West Africa, GeneXpert devices located in mobile laboratories in Guinea and Sierra Leone were interfaced to GxAlert to enable real-time collection and management of diagnostic data. GxAlert automatically reported Ebola-positive cases directly to the laboratory directors in Conakry and Freetown via SMS and email alerts to provide key decision-makers with accurate, reliable and timely information to decrease the time needed to coordinate a response.

Once the results from diagnostic instruments are digitised, several patient-centred interventions also become immediately possible:

- Faster treatment enrolment: Results can be transmitted in real-time via SMS, email, online dashboard or connection to another database (e.g. electronic patient record) for faster treatment enrolment. In Malawi, SystemOne’s Aspect platform is used to transmit HIV viral load results from the central laboratory directly to the referring clinics in real-time. Clinicians confirm receipt to acknowledge that they are acting on the results.^29^ In the context of AMR, the timely feedback of patient results supports clinicians in providing prompt patient management and decision-making on antibiotic use.^30^
- Immediate contact tracing: When results are tagged at the laboratory with patient name, phone number and address, this information can be transmitted immediately (or after approval) to contact tracing teams to contain outbreaks earlier. Training of staff to maintain confidentiality is critical for the optimal operation of such a system.
- Establishment of ‘sentinel sites’ for outbreak containment and AMR surveillance: Diagnostic positives in neighbouring countries can alert their national emergency operations centres and the Africa CDC’s RCCs. By connecting sentinel sites along borders or key points of entry, neighbouring countries can better prevent outbreaks from crossing their borders. Developing capacity for AMR surveillance and data collection at sentinel sites can help inform antibiotic prescription guidelines and infection control policies, inform intervention needs and help develop an understanding of emergence, transmission and dissemination of pathogens.^30^
- Tagging and geo-locating sample transportation: Connected, integrated sample transport will contribute to solving the problem of sample loss, delays and misplacement. Geo-tagging sample containers with similar identifiers with patient source and point of origin will rapidly improve the speed of sample access and eliminate the administrative bottlenecks of tracing missing samples.

### Operational dashboards and interoperability

A further need of the Africa CDC is to link various surveillance mechanisms to create a holistic, integrated system. Connectivity solutions are not designed to replace existing systems, but rather to feed them faster and with more accuracy than manual collection with critical data. Existing disease surveillance systems and laboratory information systems (e.g. Global Antimicrobial Resistance Surveillance System, Global Early Warning System, DISA, District Health Management Information System-2) are robust tools with well-established processes. What they need most are better sources of timely and accurate data. Connected diagnostics help these systems better fulfil their designed purpose.

Even without complex laboratory information systems, connectivity solutions provide an immediate subset of structured data that is sufficient for core activities. Programme managers, the Ministry of Health, the national reference laboratory and other stakeholders can remotely monitor reliable and accurate patient diagnostic data, rates of positive cases, trends and geospatial information depending on their different levels of access to operational dashboards. These dashboards can provide some data analytic capabilities to support interpretation and exploration of trends and anomalies, and data can also be easily aggregated to multiple levels of stakeholders for real-time decision-making and response.

## A bottom-up approach to continent-wide antimicrobial resistance surveillance

**FIGURE 1:**
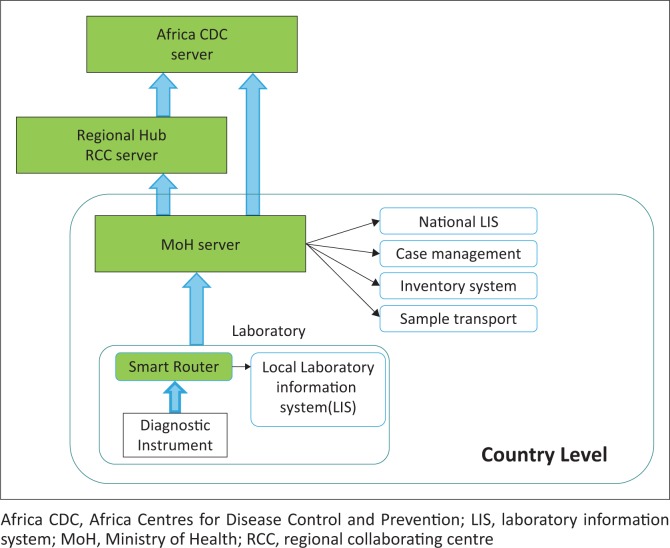
Potential flow of information between national, regional and Africa CDC. At each level, the server manages what information and triggering conditions determine further reporting.

Building disease surveillance on connected diagnostics will enable a coordinated response to potential public health threats. In a scenario where a country has diagnostic instruments connected to a system such as GxAlert or Aspect, for example, digital copies of geo-located, disease-positive cases are stored on a country-level database. Each country as well as the Africa CDC RCCs and headquarters could have access to their own operational dashboard based on the permissions set by the Ministry of Health (Figure 1). This type of system would serve the Africa CDC’s need for real-time, geospatial reporting of quality results and enable them to monitor de-identified results of importance, while still preserving the country privacy and controls in place. It would also serve to strengthen the coordinating and supporting role for the RCC in specific sub-regions.

Automated escalations from the national to the RCC level, based on certain thresholds, can initiate effective coordination and allow cross-border surveillance (Table 1). Significant value is obtained from the ability to receive real-time statistics on disease burden, outbreaks, at-risk populations and other epidemiological metrics for further public health threats.

**TABLE 1:** Bottom-up approach to a continent-wide disease surveillance network showing how access can be restricted based on permissions.

Components of a disease surveillance network	Level of access		
	Country-level	Regional Hub	Africa CDC
Surveillance	- Real-time diagnostic results - Patient demographics - Time-stamped - Geo-location of instruments and positive cases (spatial resolution)	- Trigger alert when numbers of positive cases detected exceed a predefined threshold - Number, dates, and country locations of positive cases detected (de-identified)	- Trigger alert when numbers of positive cases detected exceed a predefined threshold - Number, dates, and country locations of positive cases detected (de-identified)
Operational dashboard	- Supply chain and forecasting - Instrument utilisation rates and errors - Automated statistics and reporting - Real-time monitoring of disease progression, trends and epidemiological information - Standardised data collection linked to country health information systems	- Automated, aggregated statistics and reporting on disease burden, outbreaks and circulating strains - Ability to monitor response and impact	- Automated, aggregated statistics and reporting on disease burden, outbreaks and circulating strains - Ability to monitor response and impact
Preparedness	- Reporting system for positive cases to various designated levels of the health care system - Structured escalation system to regional hubs and central surveillance centers	- Activate in-country monitoring for disease cases - Cross-border preparedness; establish sentinel sites	- Effective coordination and communication
Response	- National response mechanism - Timely communication to mobilise case detection - Outbreak notification system	- Allocation of additional resources to countries, as required	- Monitoring of outbreaks to channel appropriate and timely resources

Africa CDC, Africa Centres for Disease Control and Prevention

## Confidentiality and privacy of health information

The protection of both patient privacy and national sovereignty are, naturally, strong concerns. The International Health Regulations^31^ provide the legal framework and political agreement needed to help the international community prevent and respond to any potential cross-border threats. However, in practice, creating an effective mechanism for sharing this critical data in a transparent and timely manner has been challenging. The advent of connected diagnostics introduces a new opportunity. The technology is capable of alerting all of the necessary organisations in real-time. The Africa CDC has the mandate and the legal underpinnings within the International Health Regulations to work in concert with nations to negotiate what levels of data access are acceptable and under what conditions (Figure 1). The RCCs do not need to see details that identify an individual patient and might not need to even see specific results, but could be alerted if more than 10 positive Lassa fever results (or plague or Ebola, etc.) occurred within a single month. The alert may simply facilitate a conversation between the RCC and the national emergency operations centre about the cases to see if resources are needed. Although a diagnostic result is not the same thing as a ‘positive patient’, the information is close enough that for pathogens of public health importance, the appearance of an unexpected number of positive results warrants a conversation between the public health bodies involved, at the minimum. The legal framework supporting this has existed for some years. The technology now exists to establish such a surveillance network very affordably; for example, based on the experience of installing and maintaining connectivity services for more than 2100 Cepheid GeneXperts, Abbott m2000s, Becton Dickinson MGITs, Alere PIMAs and Qs, and others by SystemOne, Savics and the Foundation for Innovative New Diagnostics (FIND), the cost to connect the entire continent of Africa’s HIV viral load instruments is only around $5 M per year (or $91 000 per African country). Doing so would provide the systems needed to give every country full, digital access to their HIV viral load testing results at a national, regional, district or site level, the RCC reduced and de-identified access to HIV data in their region, and the Africa CDC the real-time summary information necessary to guide effective policy. Putting the entire continent’s HIV drug resistance testing into a multi-tiered AMR surveillance network is extraordinarily inexpensive and yields extraordinary AMR insights around one of the deadliest diseases in history. The costs for connecting diagnostics for pathogens of concern is, in our opinion, at or below the cost of doing so for HIV. This is attainable in the short term.

## Conclusion

As we prepare for future outbreaks and monitoring of AMR, we should use the advanced technologies that are available to us in order to evolve and strengthen key factors in ensuring a well-functioning surveillance strategy: laboratory detection, reporting and response systems. The opportunity exists to take advantage of the ability to collect unprecedented amounts of electronic data from diagnostic platforms through connectivity solutions. By connecting the deployed devices at the beginning of an outbreak or, better yet, prior to new outbreaks during simulations of preparedness, a highly reliable and real-time system for detection and response can be activated at ‘patient zero’. Immediate reports via any electronic means of positive cases can be sent directly to key decision-makers and response coordinators, reducing the time to response, improving data quality issues and stopping unnecessary deaths due to prolonged disease transmission. Reliable, automated data, sent directly from diagnostic systems, enables national, regional, and continent-wide political and health decision-makers to act confidently, as all stakeholders will be utilising high-quality data generated in real-time.
